# Measuring Age Discrimination at Work: Spanish Adaptation and Preliminary Validation of the Nordic Age Discrimination Scale (NADS)

**DOI:** 10.3390/ijerph16081431

**Published:** 2019-04-22

**Authors:** Patricia Carral, Carlos-María Alcover

**Affiliations:** 1 Chemical engineer and Master in Job Risks Prevention, Universidad Rey Juan Carlos, 28922 Madrid, Spain; p.carralolalla@gmail.com; 2 Department of Medicine and Surgery, Psychology, Preventive Medicine and Public Health, Immunology and Medical Microbiology, Nursing, and Stomatology, Universidad Rey Juan Carlos, 28922 Madrid, Spain

**Keywords:** age discrimination at work, older workers, age stereotypes, validation, Nordic Age Discrimination Scale

## Abstract

Negative stereotypes about older workers can result in ageism and age discrimination in the workplace. The aim of this study is to carry out an adaptation to Spanish and a preliminary validation of the Nordic Age Discrimination Scale (NADS) in a sample of Spanish workers over 55 years of age. The study involved 209 employees aged between 55 and 67 years old (155 women (74.2%) and 54 men (25.8%)) working in the health sector with different professional categories (nurses, doctors, nursing assistants, ancillaries and health technicians). The reliability index of the six dimensions of the NADS (promotion, training, development, development appraisals, wage increase and change processes) measured by Cronbach’s alpha was α = 0.83. The exploratory and confirmatory factor analyses, with the goodness-of-fit indexes used, reflect an acceptable adjustment of the single-factor structure of the NADS. Regarding criterion and construct validity, the NADS correlated positively and negatively with the respective variables in the expected directions, except in one case. These results indicate that the Spanish version of the NADS shows adequate levels of internal consistency and criterion validity, and this instrument meets standard psychometric properties in its Spanish version.

## 1. Introduction

Given the increasing participation of older workers in the labour markets of developed countries [1,2] it is crucial to understand the perceptions of employers and employees regarding age at work [3,4,5]. Though perceptions of older workers have changed markedly over the last decade [6], and negative and positive beliefs coexist [7], age discrimination continues in work contexts [8,9,10,11,12]. Persistent misperceptions and negative stereotypes about age, which generally associate older workers with scant motivation, less alert capacity and limited productivity, less flexibility, more resistance to change, unwillingness to learn, less reliability for health reasons, and poor technological and digital skills [7,13,14,15,16,17], all too often contribute to an age discrimination climate in organizational settings [18]. However, most of these age stereotypes have been refuted by empirical research, or at least they can admit many nuances, and conclusions are inconclusive [15]. In this sense, attention has been devoted to the role of social processes in the creation, maintenance, and reproduction of ageism in the workplace, to the power relationships implied, and to the discursive, relational nature of ageism in general [19,20].

The term ageism is generally used to refer to the “process of systematic stereotyping and discrimination against people” because they are old ([21], p. 22). Meanwhile, age stereotypes are general beliefs and judgments about individual employees based on their chronological age rather than on their actual competencies or KSAs [22]. They are the cognitive component of a broader constellation of negative affects and emotions (that is, prejudices), behaviour intentions, decisions and social/cultural norms towards older employees and ageing. 

The triad of negative stereotypes, negative emotions and discriminatory behaviour towards older workers [23] is aligned with the distinction between ageism and age discrimination proposed by [24]. These authors distinguish between an ageist ideology, comprising both cognitive components (negative stereotypes and beliefs) and an emotional core (at the heart of negative attitudes), and age discrimination, which refers to specific behaviours that exclude or disadvantage certain people because of their chronological age. Hence, age discrimination at work occurs when exclusively age-based beliefs become the foundation for unreasonable, unfair and negative employment decisions affecting older workers [22]. Following the causal chain, age-based stereotypes can in turn produce age bias, which takes the form of errors of judgment and false assumptions caused by the general tendency to think about older workers in either positive or negative terms. The consequence may be either to favour such employees or to disadvantage them based merely on their age [22]. Age discrimination is an element of the broader concept of ageism [25,26], which entails both prejudice and employment discrimination affecting the career opportunities, access to learning and training, and job continuity of older workers [27,28].

Employment rates among older workers provide one objective measure of the possible existence of age discrimination. According to OECD figures [29], Sweden is the country with the highest employment rate (76.4%) for the 55-64 age group. The rates are 65.0% for the G7 nations, 57.1% for the USA and 57.0% for the EU 28. Despite the growth in these rates seen over the last two decades, the percentages are still lower than would be needed to ensure the participation of older people in the labour market, support active aging and underpin pensions and social protection systems, taking into account rising life expectancy and the rapid aging of the population. Far more worrying, however, is the employment rate among the over-65s. The country with the highest rate is South Korea where it is 31.5%, compared to 19.3% in the United States, 14.9% in the G7 and just 5.7% in the EU 28. Given that Europe includes several of the countries with the oldest populations in the world (Italy, Spain, Germany and France), these percentages suggest that a growing number of older people are leaving the labour market, whether voluntarily or involuntarily, while they still have many years of productive life left. As a result, the expansion of the period of life currently defined by retirement has created two age groups, which are usually demarcated at around 75–80 years of age [30]. The rate of labour market participation among older people depends on both individual factors like health, level of physical activity and control of work, which influence the decision to retire or to continue working [31] and on contextual factors like retirement legislation, social norms and organizational policies, which affect employers’ practices and decisions with regard to hiring, retention and severance of older workers [32]. Whether an individual opts to stay on or to leave the labour market is in most cases the outcome of a complex combination of factors of both kinds.

Though the phenomenon is found in all sectors of the economy [16], there is no consensus among scholars as to the causes of age discrimination. In general terms, it has been attributed to imperfections in the labour market (e.g. biased beliefs and information about the competences of older workers), the result of rational decisions (e.g. to cut labour costs by letting more expensive long-serving workers go) and the effects of non-run transformations in the nature of today’s neoliberal economy (e.g. the staid and unexciting profile of older workers compared to the vitality and innovation of the young in a highly dynamic and competitive labour market) [17]. Meanwhile, [33] summarize the key types and subtypes of workplace ageism as including positive and negative ageism [34], implicit and explicate ageism [35], personal and institutional ageism [34], interactive ageism [36], compassionate ageism [25] and gendered ageism [37].

Previous research has found that the experience of age discrimination at work is related with several negative consequences for employees. First, ageism is associated with worse health outcomes for older workers, such as lower physical or functional health [38], lower mental health and problem drinking [39], lower psychological well-being [40], and increased psychological distress [41]. In particular, [42] found in a longitudinal study that, in the case of older women workers, the negative effects on emotional health (emotional states related to clinical depression) corresponded to the most recent experiences of discrimination, while the physical health problems (functional mobility limitations) appeared several years after the discrimination was reported. Another longitudinal study [43] showed that discrimination based on age was significantly associated with poor subjective health, greater disease burden, and lower emotional health (lower life satisfaction and greater loneliness), both when measured at the outset and four years later, detecting a worsening in the course of those years.

Results also evidenced that perceptions of work ageism were related with psychological states, attitudinal and behavioral variables such as lower willingness to learn and train [44]; decreased work engagement and lower sense of organizational membership [8,45]; lower job satisfaction and organizational/affective commitment and higher actual job withdrawal [18,46,47,48]; lower self-esteem and perceived personal control [49]; lower orientation to promotion and career development [13,50]; lower desired retirement age and poor expected retirement adjustment [51]; and heightened intentions to leave job and to retire early [8,47,52]. In addition, the decision to retire prematurely as consequence of perceived age discrimination at work can adversely affect a person’s economic situation in retirement [17,53], both in subjective and objective incomes [54].

Based on these results and the variables included in previous studies on consequences of ageism at work [8,18,25,49,55,56], we selected the variables self-efficacy, perceived stress, work ability, job and life satisfaction, social support (co-workers support and supervisor support), retirement intentions, and health impairments to analyze their relationships with perceived age discrimination. It is expected that perceived ageism at work correlates negatively with self-efficacy, work ability, job and life satisfaction, co-workers support and supervisor support, and positively with stress, health impairments and early retirement intentions.

In workplace contexts, then, it is crucial to devise tools capable of measuring perceptions of age discrimination and their correlates accurately and allowing comparison between samples of older workers in different countries and across industries and economic sectors. The measurement of discriminatory attitudes and behavior towards older workers has been carried out with diverse instruments. For instance, the Workplace Age Discrimination Scale (WADS [57]) is based on older workers’ experiences of age discrimination. The Workplace Intergenerational Climate Scale (WICS [58]) evaluates attitudes and perceptions of workers pertaining at different age groups and intergenerational dynamics at work. Other measurement instruments include direct queries about being ignored by supervisors or being overlooked for promotions [10], or about perceived age discrimination climate, identifying organizational processes that could be source of potential age discrimination in the workplace, like performance assessment, allocations of tasks, professional and personal development, career opportunities, and leadership behaviors [18]. And the Nordic Ageism Discrimination Scale (NADS [25]) is created to assess the perception of age discrimination at the workplace. This scale use six items for measure six basic processes implied in work settings: promotion, training, development, development appraisals, wage increases, and change processes. Due to its unidimensional structure, its parsimonious character and its well-contrasted psychometric properties, we consider that NADS is an excellent instrument to measure age discrimination in the workplace.

The aim of this paper is twofold. In the first place, we seek to adapt and validate the Nordic Age Discrimination Scale (NADS) [25]) in a sample of older workers given the lack of reliable, properly validated instruments in Spanish, and in the second to establish whether workers’ age is related with perceptions of workplace age discrimination, with perceived inequalities in the workplace and bullying at work, and with perceived health, wellbeing, work ability, perceived social support at work, retirement intentions, and job and life attitudes.

### Context of the Study

Spain has one of the oldest populations, not only in the EU, but in the world. In 2017, life expectancy at birth was 83.1 years (the second highest among the OECD countries after Japan, 83.7 years) [59] which breaks down into an average of 85.7 years for women and 80.4 years for men [60]. In 2018, some 19.1% of Spaniards were aged 65 or older [61], and estimates suggest that the percentage will rise to 37.8% by 2051, breaking down into 21.4% of the population aged 65–79 years and 16.4% aged 80 or more [62]. The average age of the Spanish population, which is another indicator of aging, stood at 2018 in 43.1 years [61]. Meanwhile, the ageing index for 2017 was 120.4, which means there were 120 people aged over 64 years for every 100 below the age of 16 years [63].

However, the employment rate among the 55–64 age-group is only 52.5% and above the age of 65 it is a mere 2.0%. [29]. The legal retirement age in Spain is 65 years and six months, which will be progressively raised to 67 years by 2027, except in the case of individuals who can show at least 38.5 years of social security contributions. 

The combination of rapid population ageing and low rates of unemployment among people aged over 55 makes for a ticking time bomb of older workers that could have devastating consequences in the not-so-distant future. In this light, it is essential urgently to establish whether the low rate of labour market participation among people aged 55 and older in Spain is due, among other factors, to the existence of workplace age discrimination, and to do this we need adequate measurement tools to identify the factors underlying the socio-employment context. According to Article 35 of the Spanish Constitution, *“All Spaniards have the duty and the right to work.”* At no point however does the Constitution say that this duty and right lapses at a given age, or that people must retire when they reach the age of 65 or 67.

## 2. Materials and Methods 

### 2.1. Ethics Statement

This study has been approved by the Research Ethics Committee of Universidad Rey Juan Carlos (Madrid, Spain) and meets all ethical and legal standards are applicable to research of this survey modality.

### 2.2. Participants

The study sample comprised 209 health sector workers aged 55 years or older belonging to different professional categories (doctors, nurses, auxiliary nurses, healthcare technicians and orderlies). All of them were employed at various medical and health facilities located in the Madrid region (public and private hospitals, outpatient clinics, one workplace accident firm and one employment risk prevention firm).

The sample was made up of 155 women (74.2%) and 54 men (25.8%). These percentages are normal for what is a highly feminized profession. According to the Spanish Working Population Survey, 77% of public and private healthcare workers in Spain are women [59]. The sample workers were aged between 55 and 67 years with a median age of 58.6 and SD of 2.87 years. Their medium length of service to their organizations was 16 years with an SD of 12.12years.

The majority of the sample workers (66.5%) were married, 12% were unmarried, 7.7% were divorced, 6.7% were widowed and 2.4% were separated. The breakdown by professional category was as follows: nurses 34.4%, auxiliary nurses 28.7%percent, doctors 19.1%, orderlies 10.5% and healthcare technicians 5.3%. These percentages are similar to the staff breakdowns for each participating facility, and the variation was in no case greater than 10%, which means the sample reflects the professional demographics of the industry relatively well.

### 2.3. Procedure

Employees at various local and regional medical and healthcare facilities, both public and private, in the Community of Madrid were invited to take part in the study on a voluntary basis. Initially, a member of the research team provided each participant with written information about the study and an informed consent form. After reading the information and agreeing to take part in the study by signing the form, the participants were given a paper questionnaire and an envelope. After completing the questionnaire, the participants handed the sealed envelope directly to the researcher in order to guarantee the confidentiality of their responses. The data were collected in the months of March and November 2017. The response rate was 87% out of 240 questionnaires in total, of which 31 were rejected because the respondents failed to provide socio-democratic data concerning age, gender or professional category. All of the remaining valid questionnaires were fully completed and none were therefore eliminated due to missing data in the response scales.

### 2.4. Instruments

The Nordic Age Discrimination Scale (NADS) consists of six items, one for each dimension: (a) promotion, (b) training, (c) development, (d) development appraisals, (e) wage increases, and (f) change processes [25,64]. Participants were asked to indicate to what degree they agreed with each item on a five-point scale, where 1 = totally disagree and 5 = totally agree. Internal consistency of the NADS in the validation study [25] was between 0.82 and 0.87 measured in terms of Cronbach’s alpha. 

To prepare the Spanish version of the instrument, the researchers translated the six original items, which were then back-translated into English by a native speaker independent of the research team with bilingual competence in Spanish. After the equivalence of the six items in both languages was verified, the Spanish-language items were included in the general survey. Cronbach’s alpha was 0.83 in our study, which shows that the internal consistency of NADS is stable in the Spanish language version compared to the original validation, which supports the robustness of the scale.

Two further items were then included following the same procedure as used in the original validation of the scale to verify the validity of criteria. The first of these measures perceived age inequalities in the workplace (‘Have you noticed any inequalities in how older and younger [workers] are treated at your workplace?’) based on the following response scale: (1) very seldom or never, (2) rather seldom, (3) sometimes, (4) rather often, and (5) very often or always. The second item measures perceived bullying at work (‘Have you been subjected to bullying or harassment at the workplace during the last six months?’), with the same response scale. This represents a change with respect to the original scale, where this item had two response alternatives: (1) no, and (2) yes [25]. In order to maintain Likert scores in all items, we decided on this modification of the response scale. The same translation and back-translation procedures were used as for NADS. Although single-item measures are not ideal, they have been used successfully in research to examine the negative effect of race, weight and sex discrimination on several individual variables [43]. Meanwhile, there are areas of research and constructs relevant to occupational health psychology that have been assessed successfully with single-item measures, including bullying at work, stress symptoms, perceived depression, life satisfaction, or job satisfaction, among others [65]. In addition, [66] argued that single-item measures are more applicable and acceptable when constructs are concrete, one-dimensional, or have high semantic redundancy, characteristics that concur in the constructs measured with a single-item in this study. 

Following the procedure used in the original validation of the instrument [25], different correlates of age discrimination were measured, whose relations with the NADS permitted testing of the construct validity:

*Self-efficacy* was measured from the QPS-Nordic ADW [64]. The scale has four items that use a Likert scale of five response points (1 = totally disagree, 5 = totally agree). Examples of items are: “I can manage what I do at work as good as others” and “I have the capacity to handle most of the situation in my work”. In Nordic samples, the scale showed an index of reliability of α = 0.78, and in this study Cronbach’s alpha value was 0.66.

*Perceived Work ability*. This variable was measured from the Nordic Questionnaire for Monitoring the Age Diverse Workforce questionnaire (QPS-Nordic ADW) by [64] and from the Spanish adaptation made by [67] of the short version of the Work Ability Index (WAI) [68]. The scale had five items that described perceptions regarding work ability. Examples of items are: “Your work is...:” with the response alternatives: mentally demanding; physically demanding; mentally and physically demanding; or “How do you rate your current work ability with respect to the mental demands of your work?” with the response alternatives: (1) very poor; (2) rather poor; (3) moderate; (4) rather good; and (5) very good. In this study, the Cronbach’s alpha value of the three Likert items was 0.78.

*Stress*. This variable was also measured from the QPS-Nordic ADW [64]. The construct was measured with a single-item: “Stress means the situation when a person feels tense, restless, nervous, or anxious, or is unable to sleep at night because his or her mind is troubled all the time. Do you feel that kind of work-related stress these days?” With the response alternatives: (1) not at all; (2) only a little; (3) to some extent; (4) rather much (5) very much.

*Job and life satisfaction*. This variable was measured from the QPS-Nordic ADW questionnaire by [60]. The construct has three items “How satisfied are you with your present work?” “How satisfied are you with your present life?” Response scale range from (1) very dissatisfied, to (5) very satisfied. And “Do you feel positive about how your work will develop in the future?” Response scale range from (1) not at all, to (5) very much. In Nordic samples the scale has shown an index of reliability of α = 0.68, and in this study the value of Cronbach’s alpha was 0.59.

*Social interaction* was measured through two variables used in the QPS-Nordic ADW [64], perceived co-workers support and superior support, with three items each one. Response scale range was: (1) very seldom or never, (2) rather seldom, (3) sometimes, (4) rather often, and (5) very often or always. Examples of items are: “If needed, are your co-workers willing to listen to your work- related problems?”, and “If needed, can you get support and help with your work from your nearest superior?” In this study, Cronbach’s alpha of co-workers support subscale was 0.86 (α = 0.84 in Nordic samples), and Cronbach’s alpha of superior support subscale was 0.89 (α = 0.91 in Nordic samples).

*Retirement intentions* were measured with the scale developed following the procedure of previous studies [67,68,69,70] and formed by five statements regarding three possible different pathways for retirement,: full retirement (“As soon as I can retire, I will definitely stop working”); part-time retirement (“Even when I can already retire, I will keep on working but reduce my actual work effort in terms of working time”); late retirement (“Even when I can retire, I will keep on working”); early retirement (“I will retire early if possible”); and bridge employment (“Even when I can retire, I will keep on working but changing job type”). Participants were asked to reflect on the future and to state their degree of agreement or disagreement on a five-point scale (where 1 indicated “completely agree” and 5 “completely disagree”). Participants were asked to think about their future and declare their retirement intentions stating their degree of agreement or disagreement with the three items on a five-point scale (where 1 indicated “completely agree” and 5 “totally disagreed”). In this study, Cronbach’s alpha of retirement intentions was 0.66.

Finally, *health impairments* were estimated from the self-reported days of job sick leave during the last year: “How many work days during last 12 months have you been absent from work because of your own sickness?” Response range: 0 days, 1–7 days, 8–24 days, 25–99, 100 days or more.

### 2.5. Data Analysis

**Figure 1:**
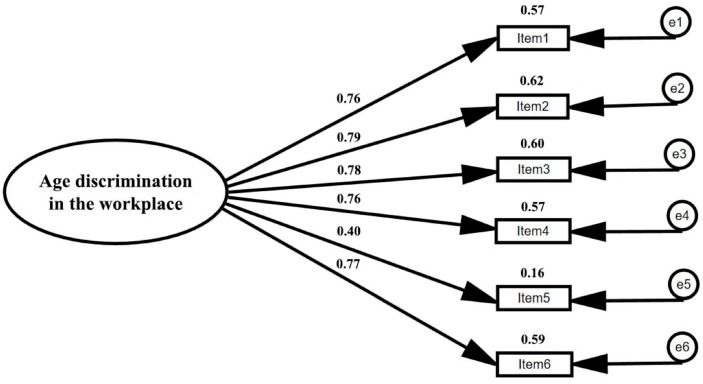
Single factor model of age-based discrimination in the workplace by NADS.

In the first place, the unifactorial structure of the NADS was compared through the use of factor analysis procedures. The sample (*N* = 209) was divided in half, applying exploratory factor analysis (*N* = 104; Table 1), and confirmatory factor analysis (*N* = 105; Table 2). In order to analyze convergent validity, our analysis is based in the criterion that item-factor saturations should be higher than 0.70, with consideration given to how many saturations are significant [71] (Figure 1). We then tested reliability (Cronbach’s Alpha) and frequency descriptives (mean, SD, range), and we finally ran correlation analyses to test criterion validity and construct validity (Table 3).

## 3. Results

To test factor structure, an exploratory factorial analysis (extraction method: principal component analysis, with varimax rotation) was conducted on half the sample (*N* = 104) with the six Spanish version NADS items loaded in a single factor (Table 1).

With regard to confirm the factorial structure, a confirmatory factor analysis was also performed with half of the sample (*N* = 105). Again, the six Spanish version NADS items loaded in a single factor. Confirmatory factor analysis (Maximal Likelihood; Eigenvalues ≥ 1) found factorial weightings in a range of 0.40 and 0.79. The goodness-of-fit indexes used (Table 2) reflect an acceptable fit of the single-factor structure of the NADS: *χ^2^* = 15.716; *df* = 9; ratio *χ^2^*/*df =* 1.746; RMSEA = 0.085; CFI = 0.974; TLI = 0.939 (low values of 3 for ratio *χ^2^*/*df*, low values of RMSEA 0.08, and values of CFI and TLI in the range of 0.90–0.95 suggest an acceptable fit; [72]). These results for factorial weightings are similar—they were slightly lower in our sample– to those obtained by reference in the original in NADS validation, both for the total sample and for the three sub-samples (Norway, Sweden and Finland; [25]).

Regarding convergent validity analysis (Figure 1), all item saturations from corresponding factors were significant (*p* < 0.001), ranging between 0.40 and 0.79, fulfilling requirements for convergent validity [71].

In terms of the scale’s internal consistency, Cronbach’s alpha was found to be 0.83, showing the robustness of NADS. The internal consistency of NADS in the validation study [25] ranged between Cronbach’s alpha 0.82 and 0.87, and the value obtained for the Spanish sample is therefore in the same range.

With regard to criterion validity, our results showed a positive and statistically significant correlation with the item (*r* = 0.55, *p* < 0.01) based on perceptions of age inequality in the workplace, which means the participants do not consider that there is any noticeable difference in the treatment of older and younger workers at work. The correlation was *r* = 0.48, *p* < 0.001 in the original validation by [25]. Harassment and bullying were also positively correlated with age discrimination (*r* = 0.26, *p* < 0.01; *r* = 0.23, *p* < 0.01, in [25]). These results support the criterion validity of the item utilized in the Spanish version of the age discrimination scale. Table 3 shows the descriptive results for the variables and the correlation values.

Related to construct validity, it was assessed by correlating the age discrimination scale to seven variables described above: self-efficacy, work ability, perceived stress, job and life satisfaction, co-workers support, superior support and sickness absence. Based on the empirical evidence described in the Introduction, it is expected that older workers who perceive high levels of age discrimination at work will also report higher levels of stress and lower levels in all scales of the variables analyzed. Age discrimination was positive and significantly correlated with job stress (r = 0.31, *p* < 0.01) and job sick leave (r = 0.16, *p* < 0.05). Inversely, NADS correlated negatively and significantly with self-efficacy (r = −0.22, *p* < 0.01) perceived work ability 1 (r = −0.19, *p* < 0.01, respect to three-items subscale; and related to the item “Current work ability compared with the lifetime best. Assume that your work ability at its best has a value of 10 points. How many points would you give your current work ability?”, correlation of work ability 2 with NADS was r = −0.18, *p* < 0.01), job and life satisfaction (r = −0.20, *p* < 0.01), perceived co-workers support (r = −0.31, *p* < 0.01), and perceived superior support (r = −0.34, *p* < 0.01) (Table 2). Finally, no correlation was found between NADS and retirement intentions (r = 0.049), when we expected that low age discrimination perception was associated with lower early or full retirement intentions.

## 4. Discussion

In light of recent data reflecting and appreciable increase in age discrimination at work, it is urgent that reliable valiant tools be found to measure this pervasive phenomenon [10,25]. Furthermore, quantitative instruments are needed to obtain a more objective estimation of the scale of the phenomenon. These tools need to be adapted for application in different national and language contexts, and the preliminary validation presented here offers support for the adequacy of the psychometric properties of the Spanish version of NADS.

Having a reliable and valid instrument to measure age discrimination can be very useful for making diagnoses and for subsequent design of organizational interventions for preventing an older worker’s discrimination. As recently pointed out by [73] (p. 138): “perhaps the greatest need in this area is to be able to provide organizations with best practices for reducing discrimination”.

The results obtained in this study allow us to conclude that the Spanish version of NADS presents a factorial structure equivalent to the original scale and adequate levels of reliability, as well as to check its criterion validity and its construct validity. In sum, the Spanish version of NADS presents adequate psychometric properties for its use in ageing-at-work research. In addition, our results are coincident with extant research evidence on the relationships of age discrimination at work and several negative consequences for older employees, as we have revised in the Introduction of this paper.

The impact of ageism in the workplace can be observed in increasing age-related discrimination claims, increasing HRM practices oriented to disparate treatment of older and younger workers regarding important workplace decisions such as recruitment and hiring choices, differences in treatment in access to training and development opportunities, and (negative) judgments of potential for advancement in mid and late career [1,74,75,76]. Ageism at work can also be observed in decreasing organizational retention, increasing job leave decisions, forced retirement, unemployment and increased time for older people to find employment [7,74]. These prevalent forms of age discrimination at work represent serious barriers to employment, health and financial wellbeing in later life [77]. Consequently, age discrimination must be taken seriously and must be measured so that the real dimension of this negative phenomenon can be understood. Although NADS provides a subjective (perceptive) measure of age discrimination, and may be affected by social desirability in the responses of participants [25], it is essential for an approach to the older worker’s job experiences. Future studies should triangulate the data sources and compare perceptions of age discrimination of older employees with those of their younger colleagues and those of their superiors and other organizational agents.

NADS could be used to analyze the relationships between perceived age discrimination at work and socio-demographic variables such as gender, professional category, seniority in the organization, contract type and so on, as well as associations with variables related with health (e.g., burnout, cardiovascular disorders), work-related personal resources like psychological capital and meaning-making [78], and organizational climate [18]. Future studies using this instrument could also establish the strength of these relations and allow researchers to hypothesize and delve into the potential effects of perceived age discrimination at work as a key factor in early retirement decisions [79], later mobility and bridge employment intentions [63,80], and the physical and mental health and well-being of older workers, their responses to stress and their involvement in unhealthy activities [81]. The fact that our results have not shown a relationship between age discrimination and retirement intentions may be due to the characteristics of the sample, formed by health professionals. For these employees, retirement, at least in the public sector, is highly regulated by chronological age or tenure/years of service. Future studies should use workers from other professions to identify possible relationships between these variables.

On the other hand, our results generally confirm the relationships between age discrimination and the outcome variables analyzed. Thus, we found that age discrimination at work was positive and significantly correlated with job stress and job sick leave, which shows its relationships with worse well-being and perceived health; these results are consistent with those obtained in previous studies, confirming that perceived age discrimination represents a work stressor [25,48,82,83], and does impact on subsequent health [42]. Inversely, age discrimination correlated negatively and significantly with self-efficacy, work ability, job and life satisfaction, perceived co-workers support, and perceived superior support. Previous studies have shown the negative relationship between age discrimination and perception of fewer resources at work (e.g., efficacy, competencies, learning ability and so on) [25,51]. In addition, as consequence of ageism at work, perception of limited opportunities for promotion, training, and development reduce skills and competencies (leading to lower perceived work ability), work motivation, and the—subjective and objective—employability of older workers [84]. In a similar vein, there is strong evidence on the negative relationship between perceived age discrimination and psychological wellbeing at work, organizational commitment, and job satisfaction [25,40,48,55,85,86,87,88], in line with results obtained in our study. Finally, regarding social factors at work, our results coincide with other studies [8,10,25,88], showing a negative relationship between perceived age discrimination and perception of social support; this negative relationship is significant both in relation to the supervisor and to coworkers, which shows that age discrimination is vertical and horizontal. These results are revealing of the need to devote more attention to promote intergenerational contact and relationships at work, and to adopt an organizational multi-age perspective [89].

This study presents the usual limitations of a cross-sectional design based in self-reported measures in organizational research [90], although it is possible, as [91] suggested, that the problem derived from the action of common method variance is overstated. Another limitation is related to the single-item measures; although it was already argued in the Method section that they may be valid under certain conditions, future studies should avoid these single-item measures. The sample consisted of professionals from a single occupational sector (health professionals with different job qualifications and status), so future studies using the NADS should be done with other professionals working in different occupations. In addition, this dataset could be expanded and supplemented by data obtained from other national samples obtained in Spanish-speaking Latin American countries using NADS to measure the variables identified. The dataset would also make it possible in the future to create a map of perceived age discrimination at work and its consequences in different national contexts, as well as allowing the possibility of cross-cultural studies using data obtained in Europe, North America, Australia, Japan, etc., where population ageing, the rising participation of older workers in the labor market and the need to extend working life are fast becoming social, economic and political priorities [92], and where the identification, measurement and prevention of perceived age discrimination at work is a key issue for both academic researchers and professional practice.

## 5. Conclusions

In the last two decades there has been an increase in the participation of older workers in the labor market. A consequence of the greater presence of employees over 50 years has been the increase in age discrimination at work. In this context, it is necessary to have reliable and valid instruments to measure the true extent of age discrimination in organizational settings. This study aimed at the Spanish adaptation of the Nordic Age Discrimination Scales (NADS; [25]) using a sample of 209 workers aged over 55 years. The results obtained present a one-dimensional factorial structure equivalent to the original scale and adequate levels of reliability. In a similar way, criterion validity and construct validity are demonstrated, with correlations statistically significant with the proposed variables in the expected directions. In conclusion, NADS presents adequate psychometric properties for its use in research in Spanish organizations, so this instrument, already tested in Swedish, Finnish, Norwegian and English, makes it possible to obtain a robust measure of age discrimination at work.

## Figures and Tables

**Table 1:** Exploratory factor analysis of the Spanish version of the NADS, factor loading for the sample (*N* = 104).

NADS Items. Spanish [English]	Total Sample
1. Los trabajadores de edad avanzada son pasados por alto/excluidos en casos de promoción o reclutamiento interno [Elderly workers are passed over/left out in cases of promotion or internal recruitment]	0.77
2. Los trabajadores de edad avanzada no tienen igualdad de oportunidades de formación durante el tiempo de trabajo [Elderly workers do not have equal opportunities for training during work time]	0.77
3. Se prefiere a los trabajadores jóvenes cuando se introducen nuevos equipos, actividades o métodos de trabajo [Younger workers are preferred when new equipment, activities or working methods are introduced]	0.80
4. Los trabajadores de edad avanzada participan menos a menudo en evaluaciones de desarrollo con sus superiores que los trabajadores más jóvenes [Elderly workers less often take part in development appraisals with their superior than younger workers]	0.72
5. Los trabajadores de edad avanzada tienen menor incremento salarial que los más jóvenes [Elderly workers have less wage increase than younger workers]	0.52
6. No se espera que los trabajadores de edad avanzada tomen parte en procesos de cambio y nuevos métodos de trabajo en el mismo grado que sus pares más jóvenes [Elderly workers are not expected to take part in change processes and new working methods to the same degree as their younger peers]	0.78
Eigenvalue	3.2
Explained variance	54.1%

Note: N = 104. Extraction Method, principal component analysis, only one factor was extracted.

**Table 2:** Results of the confirmatory factor analysis for the Spanish adaptation of the Nordic Age Discrimination Scale (NADS).

NADS Model	*χ^2^*	*df*	Ratio *χ^2^/df*	RMSEA	CFI	TLI
1 factor	15.716	9	1.746	0.085	0.974	0.939

Note: *N* = 105; RMSEA = Root Mean Square Error of Approximation; CFI = Comparative Fit Index; TLI = Tucker-Lewis Index.

**Table 3:** Means, SD, and correlations between NADS and criterion and construct variables.

Variables	Mean	SD	NADS
1. NADS	2.37	1.07	----
2. Age inequality	2.16	1.16	0.55 **
3. Bullying at work	1.12	0.33	0.26 **
4. Self-efficacy	4.43	0.71	−0.22 **
5. Work ability 1	4.07	0.63	−0.19 **
6. Work ability 2	8.36	0.140	−0.18 **
7. Stress	2.34	1.10	0.31 **
8. Sickness absence	1.72	1.06	0.16 *
9. Job and Life satisfaction	3.70	0.71	−0.20 **
10. Co-workers support	3.99	0.88	−0.31 **
11. Superior support12. Retirement intentions	3.604.05	1.060.99	−0.34 **0.05

Note: *N* = 209; * *p* < 0.05; ** *p* < 0.01.
